# Supplementary material for: Experiencing one’s own body and body image in living kidney donors–A sociological and psychological study
Source: PLoS One. 2021 Apr 15;16(4):e0249397. doi: 10.1371/journal.pone.0249397 (PMC8049271; doi:10.1371/journal.pone.0249397)
Supplement: S1 Appendix — (DOCX) [file pone.0249397.s002.docx]

Katarzyna Kowal, PhD

Faculty of Health Sciences

Jan Długosz University of Humanities and Natural Sciences in Czestochowa

***Dear Madam/Sir,***

*My name is Katarzyna Kowal, I am a medical sociologist conducting sociological research on organ donation and transplantation. As a member of the interdisciplinary research team led by Prof. Artur Kwiatkowski (Institute of Transplantology, Medical University of Warsaw), I undertook a research project devoted to the social and psychological aspects of the donation experience.*

*I hereby kindly request you to participate in a sociological study, which will take the form of an in-depth interview. Participation in the study is voluntary. You have the option to resign from participation in the study without giving a reason, or to terminate the interview at any time during its duration. As the person carrying out this research, I ask you to take all the questions seriously and to provide honest and comprehensive answers. Your participation in the study is anonymous, and all the information obtained therein will be used solely for research purposes. I would also like to assure you about legal protection of the materials collected during the study, which will not be shared with anyone. Their publication will only take the form of quoting excerpts of statements that, in accordance with the principle of confidentiality, will not allow you to be identified as a study participant.*

*The administrator of the data collected during the interview along with your personal data is Jan Dlugosz University in Czestochowa - in accordance with the Regulation on the Protection of Personal Data (GDPR) of the European Union.*

*Thank you very much for agreeing to participate in the study!*

**In-depth interview with a kidney donor**

*The beginning of the conversation was preceded by a request to record the conversation with the use of a voice recorder.*

**QUALITY OF LIFE AFTER THE DONATION**

1. What changes happened in your life after donating the kidney?
2. How did you adapt to life after donating the kidney?
3. What do you include among the most important effects of donating a kidney?
4. How do you evaluate your health after donating the kidney? Please relate this assessment to your state of health before the donation.
5. How do you rate your physical well-being after kidney donation? Please describe it.
6. After the donation, were there any complaints related to your physical well-being that affected your quality of life? If so, please describe them. How do you feel they are related to the donation?
7. How do you rate your mental well-being after kidney donation? Please describe it.
8. After the donation, were there any complaints related to your mental well-being that affected your quality of life? If so, please describe them. How do you feel they are related to the donation?
9. How do you evaluate your professional activity after kidney donation? Please describe it.
10. Did problems in your professional sphere arise after the donation that influenced your quality of life? If so, please describe them. How do you feel they are related to the donation?
11. How do you evaluate the relations in your family after kidney donation? Please describe them.
12. After the donation, were there any problems in the sphere of family relations that affected your quality of life? If so, please describe them. How do you feel they are related to the donation?
13. How do you rate your everyday non-work activities (household chores, pursuit of passions and interests) after donating the kidney? Please describe them.
14. After the donation, were there any problems with your everyday non-work activities that affected your quality of life? If so, please describe them. How do you feel they are related to the donation?
15. How do you evaluate your contacts with other people (friends, acquaintances) after donating the kidney? Please describe them.
16. Were there any problems with your contacts with other people after the donation? If so, please describe them. How do you feel they are related to the donation?
17. Which of your post-donation problems turned out to be the most severe? How do you deal with these problems? Do you use the support of other people? If so, who?
18. Is there anything you were forced to give up after donating the kidney? If so, please indicate what it is.
19. What do you include among the most important costs related to kidney donation?
20. What do you include among the most important benefits of kidney donation?
21. What was your mood like in the first days and weeks after the donation? What moods followed?
22. Were you (donor and recipient) in the same room after the transplant? How do you evaluate this organizational solution?
23. How do you feel today, knowing that you became a kidney donor and saved the recipient's health?
24. How do you, in retrospect, evaluate your decision to donate a kidney? Was it right? Have you ever regretted this decision? If so, in what circumstances?
25. How did the process of taking the decision to donate the kidney to the recipient look?
26. How did you feel about your relative waiting for an organ from a deceased donor?
27. What situation could make you regret the decision to donate your kidney?
28. What would you say to a person considering the decision to donate a kidney and needing an advisory voice?
29. How do other people (family members, friends, acquaintances) react to your kidney donation? What are their reactions to this information?
30. How do the doctors you contact respond to your kidney donation? What are their reactions to this information (e.g. GP)?
31. Has the donation of a kidney ever been a ground of discrimination in your life? If so, under what circumstances did this happen?
32. What kind of memories do you have about the kidney donation event?
33. What moment of this event, which was the family transplant, do you recall as the worst?
34. What moment of this event, which was the family transplant, do you recall as the best?
35. Do you like to think about this event? Please justify your answer.
36. Do you like to talk about this event? Please justify your answer.

**EXPERIENCING YOUR OWN BODY**

1. What is the body for you?
2. What changes occurred in your body after the kidney donation? Please describe them. To what extent are they related to the donation of the kidney?
3. Were there any changes in the appearance of your body after donating the kidney? If so, please describe them. To what extent are they related to the donation of the kidney? What are your feelings about these changes?
4. How do you evaluate the changes that occurred in the body after the donation? (positive or negative?)
5. In which area are these changes most pronounced (body appearance or functionality)?
6. Which of these changes, if there were such a possibility, would you like to avoid?
7. Do you like your body after kidney donation? Please refer to the situation before the donation.
8. Do you take care of your body after kidney donation? If so, what does this care refer to (physical activity, dietary regimes)?
9. What bodily limitations are associated with kidney donation? How did your body restrict you in the first days and weeks after the donation? What restrictions followed?
10. What are your body limitations at present? How do you deal with these limitations? Which of these limitations do you assess as the most severe?
11. Does the appearance of the body matter to you? If so, how is it manifested?
12. How do you react to the postoperative scar resulting from the donation?
13. Are there any situations in which your scar bothers you? If so, what are they?
14. What does the issue of revealing your body in public space look like (beach, swimming pool)? In such situations, does the scar remain a problem for you?
15. How do you see your body after you donated your kidney? How do you feel about it?
16. How do you recall the pain associated with the kidney donation procedure? How would you describe this experience?
17. Which part of the body is the cause of your greatest satisfaction? Please justify your answer.
18. Which part of the body is the cause of your greatest dissatisfaction? Please justify your answer.
19. How do you feel knowing that one kidney is missing in your body? Please describe this experience.
20. How do you feel knowing that your kidney is in the recipient's body? Please describe this experience.
21. How do you think about this kidney? Do you still own the kidney or is it already the recipient's kidney?

**PERSONAL IDENTITY**

1. What kind of person do you feel after donating the kidney?
2. What changed in you as a person after donation of the kidney?
3. Were there any changes in your female/male identity after the donation? If so, what are they manifested in?
4. Were there any changes in your self-esteem? If so, please describe them.
5. Were there any changes in your behavior after the donation? If so, what are they?
6. Were there any changes in your attitude towards people after the donation? If so, what are they?
7. What is the most important value in your life for you now after the moment of donation?
8. Were there any changes in your system of values after the donation? If so, what are they?
9. What kind of person do you feel after the donation - healthy or sick? Please justify your answer.
10. What do you think your health situation will look like in the future?
11. What are your dreams and plans for the future?

**DEMOGRAPHICS**

1. **Sex**
   1. woman
2. man
   1. **Age** (please indicate your age on your last birthday)......................years old
3. **Date of organ procurement**..........................................................
4. **Who is the recipient to you?**..............................................
5. **What is your marital status?**
   1. single
      1. married
      2. separated
      3. divorced
      4. widowed
      5. other situation (what?)...........................................................................................
6. **What is your level of education?**
   1. elementary
      1. vocational
7. secondary
8. post-secondary
9. **Your place of permanent residence:**
   1. village/countryside
   2. town to 50 000 inhabitants
   3. town over 50 000 to 100 000 inhabitants
   4. city over 100 000 inhabitants
10. **What is your current professional situation?**
11. I run a farm
12. I work in a state-owned (local government) company
13. I work in a private company
14. I run my own company
15. I do not work professionally, I am unemployed
16. I do not work professionally, I'm retired, a pensioner
17. I do not work professionally, I'm a pupil, a student
18. other answer (what?).......................................................................................
19. **Are you:**
    1. a practicing believer
    2. a non-practicing believer
    3. a non-practicing non-believer (please skip question 10 of the demographics)
    4. practicing non-believer (please skip question 10 of the demographics)
    5. other answer (what?).......................................................................................
20. **What is your religion?**................................................................................
