# Supplementary material for: Experiencing one’s own body and body image in living kidney donors–A sociological and psychological study
Source: PLoS One. 2021 Apr 15;16(4):e0249397. doi: 10.1371/journal.pone.0249397 (PMC8049271; doi:10.1371/journal.pone.0249397)
Supplement: Attachment — Submitted filename: Response to Reviewers.docx [file pone.0249397.s003.docx]

30 October 2020

**Rebuttal letter**

Manuscript Number: PONE-D-20-15970

Full title of submitted article: **Experiencing one's own corporeality and body image in living kidney donors - a report from interdisciplinary studies**

Order of Authors: Katarzyna Kowal (corresponding author), Mateusz Zatorski, Artur Kwiatkowski

*Thank you for your thorough and substantive evaluation of our article. Thank you for the received comments; we were able to make significant corrections in the text. This applies to both the level of the language used and the substantive assumptions.*

*I convey the authors’ answers to the Reviewers’ comments. We wanted our responses to be as precise as the Reviewers' comments, therefore, the responses have been presented in the form of a table containing references to specific comments of the respective Reviewers.*

| **Response to comments from Reviewer No. 1** | |
| --- | --- |
| **Reviewer's comments** | **Authors' replies** |
| **Abstract**  1. "Both the methodology used and the interdisciplinary nature of the research are definitely innovative" - This needs to be moved to the conclusions as it is a statement about why the authors feel their paper is contributing to the literature.  2. Whilst the sociological perspective is not common within this field, it is by no means unique and has been incorporated into other studies. | 1. and 2.  Thank you for your comments on the content of the abstract. They all contributed to its present, much more perfect version. |
| **General comments:** 3. The English throughout the paper (and in particular the introduction) is not of the standard expected for publication in an international journal. The authors need to work on the paper considerably to improve this.  4. I was unable to access the data stated to be within a dropbox folder as the link was invalid. If you feel this data (or some of it) is relevant then it ought to be placed within a supplementary file accompanying the paper online.  5. The paper feels very long and I have made some suggestions below to bring it down.  6. I think the findings are of interest but the conclusions reached are possibly too focussed on body image; inadvertently addressing the other factors that are interplayed within this complex group of patients. | 3. Thank you for this remark. An editor specializing in scientific translations worked on the linguistic layer of the text.  4. We are sorry for this situation. Attached, as a file of supplementary information, we have presented an anonymized set of quantitative data (in the form of Exell database) collected in the psychometric part of the research, in order to potentially replicate the research results.  5. The article has been shortened in all the places indicated by the reviewers. Thank you very much for this remark.  6. The conclusions were divided into two parts, which correspond to the scope of the research issues in the sociological (qualitative) and psychological (quantitative) part of the research project. Therefore, in the first part of the conclusions we presented the conclusions from the research on the way of experiencing their own body by kidney donors, and then in the second part of the conclusions about body image. |
| **Introduction:** 7. Page 3: Second paragraph, last sentence: the word ‘his’ should be changed to ‘their’ in order to make this gender neutral.  8. Page 4, paragraph 2: I was very unclear what you were trying to say in the second half of this paragraph. I don’t believe you have searched for a new methodology – you have possibly attempted to approach the topic of body image by utilising mixed methods or a qualitative approach, but this is not really a new methodology.  9. Page 4: third paragraph, second sentence: You say ‘magazines’, do you mean journals?  10. ‘diagnose the consequences of donation’ – I’m not sure this is phrased correctly.  11. The second half of this paragraph again slips into the remit of the discussion. You need to justify why you chose this method rather than state what it allowed you to do as part of your analysis. Again this paragraph is poorly written and needs to be completely rewritten.  12. Page 4/5: When stating the study aims you need to be clear at the beginning by saying ‘the first aim of the study was x, the second aim was y’. ‘meeting the second purpose of the study’ is the wrong phraseology.  13. Page 5, paragraph 2: ‘The psychometric study provided a different kind of data’ – again you need to say that this is a mixed methods study with a qualitative and quantitative component and you need to justify why you have adopted this approach, and which part provides which data. 14. The final sentence on page 5 makes no sense. I would say that these different areas overlap, rather than them being bordered by each other. | 7. Thank you for pointing out the linguistic errors. They have all been improved.  8. Thank you for this extremely substantive remark. We fully agree that our methodology was not innovative, but simply mixed.  9. Thank you for pointing out the linguistic errors. They have all been improved.  10. Thank you for this remark. We agree with the incorrectness of this wording, which has been corrected.  11. Our analytical "going forward" instead of focusing on the motives for choosing the grounded theory methodology resulted from the fact that we wrote this part of the text at the end. The Reviewer's remark is correct, and we fully agree with it. Amendment consisting in redrafting was introduced.  12. We made corrections according to the Reviewer's suggestions. They concern not only the form (changing the place of presenting the research question and research goals), but also the content (clarification of the first research goal). We admit that the Reviewer's comment was very valuable and allowed us to increase the precision of the description and obtain greater transparency of the Introduction. Thank you for pointing out the phraseological error. It has been removed from the content.  13. In line with the Reviewer's remark, we emphasized once again the mixed nature of our methodology, explained the reasons for adopting such a methodological approach and indicated which part of the study provides which data.  14. Thank you for this remark. This unfortunate sentence has been removed. |
| **Methods:** 15. You need to state that this is a mixed methods study incorporating qualitative interviews analysed by grounded theory and a questionnaire which has provided quantitative data. Again, this methodology is less common in this field but this study is not unique in utilising this approach  16. Table 1: Employment status – please say ‘employed / unemployed’ as active / passive is unclear  17. I don’t think it is necessary to detail the entirety of the donation operation. A simple explanation would suffice and may benefit from a diagram if you wish to show where the scars are located.  18. Page 9: Examination procedure: I am unsure what ‘no masking instructions’ means.  19. Page 10: Under qualitative methods – I would not call this ‘sociological’ as it is not unique to sociology.  20. I think ‘intensive’ interview should perhaps be an ‘in-depth’ interview as this is how we would refer to it in English. The word ‘intensive’ sounds more aggressive than an in-depth interview, which as you say aims to thoroughly examine the views of the participant through focussed conversation.  21. Page 11: Final paragraph: How did you reach the conclusion that donors opened up more during telephone interviews rather than in person? This has not been the experience of a lot of participants in other studies to my knowledge. Also, you are not able to guarantee that the interview has been conducted in complete privacy as you are unclear as to who else is close by to the interviewee at the time of the interview. I know you mention that you asked the participants to consider these issues but do you have a guarantee that the optimum conditions were met?  22. I also understand the desire not to have patients attend the hospital but I am unclear how conducting it in their home deprives the study of confidentiality. Please can you explain?  23. The description of both the qualitative and quantitative methods is way too long and could be better summarised. | 15. In the text, we have repeatedly emphasized the mixed nature of the methodology used in the research and removed the content suggesting the unique nature of our research.  16. We made the suggested changes to the table regarding the employment status of the surveyed donors. Thank you for this comment.  17. Thank you for this remark. We have resigned from the detailed description of the kidney donation procedure in favor of a simple and concise explanation of what this procedure is about, the differences between the two methods of kidney donation as well as the anatomical and physiological consequences of donation.  18. In response to the reported difficulty in interpreting the concept of "no masking instruction", as well as the Editor's comments, the part of the text entitled "Examination Procedure" was supplemented with a full description of the steps leading to informed consent to participate in the study. We hope that this will increase the transparency and show the importance we attach to the ethical side of research.  19. Firstly, we resigned from the title of "Qualitative Methods - Sociological ", which in its unfortunate wording could suggest that qualitative methods are equated by authors with sociological ones and should be treated as such. We are aware that qualitative methods are not unique to sociology and are also used by representatives of other humanities and social disciplines such as psychology, pedagogy or ethnology. However, this does not change the fact that the first author of the text and at the same time the researcher of this part of the research project conducted sociological research because she consistently used the methodology of the theory in its sociological version, reaching for the interpretative social constructivism of Kathy Charmaz, who draws her methodological inspirations from pragmatism and symbolic interactionism (humanistic sociology, alternative understanding) and phenomenology.  20. We agree with this remark. Since the intensive interview as a method of collecting data in the constructivist variant of K. Charmaz's grounded theory methodology is essentially the same as the in-depth interview, we opted for the consistent use of the term “in-depth interview”. In fact, it is used more often in both English and Polish literature on the subject. We also explained in the text why we had used the term "intensive interview" when describing the method of data collection. We considered this explanation indispensable.  21. In response to the Reviewer's question about the findings regarding greater openness of the respondents during the interviews conducted by telephone, we would like to emphasize once again the fact of conducting the pilot research. It is on its basis that we established that in-depth interviews conducted by contact via telephone contain more data being a so-called dense description. The narratives of the subjects were more detailed than when the researcher met the subjects face to face. According to the researcher, this was primarily because the subject of the interview focused on kidney donation as an intimate life experience. Based on the comparison of the rhythm and content of the conversations with kidney donors made over the phone and in person, the researcher concluded that the telephone conversation gives the respondents a greater sense of psychological comfort, which results in greater freedom of expression. Without seeing the researcher, the kidney donors did not feel embarrassed to share sometimes very intimate details about their bodies. Of course, the researcher realized that in communicating with kidney donors via telephone he loses the possibility of observing their non-verbal speech, which according to A. Mehrabian's rule constitutes 555 of communication. In fact, during the telephone conversation, the researcher did not notice the respondents' body language, although she did feel it at many times. We would also like to strongly emphasize that the interviews carried out in the sociological part of the research were not telephone interviews (CATI), but in-depth interviews (IDI) conducted via the telephone. This formula of in-depth interviews was also determined by the high costs of direct interviews. Due to the large dispersion of the study participants, the costs of reaching each of them were impossible to cover by the research team. We did not have any grant for this research project.  Of course, the researcher could not be absolutely sure that no other people were present during the interview. However, she became more certain on listening to the content and course of the conversation (also while listening to the recordings). The researcher also referred to her own intuition and counted on the elementary honesty of the respondents.  22. Conducting the study at the respondent's home significantly violates his privacy because the researcher knows the home address of the research participants (sensitive data), which is contrary to the principle of research anonymity.  23. The description of the qualitative methods has been shortened so that the contents remain in line with the criteria for reporting qualitative research on the COREQ list recommended by PLOS ONE:  <https://journals.plos.org/plosone/s/submission-guidelines#loc-qualitative-research>  <https://academic.oup.com/intqhc/article/19/6/349/1791966> |
| **Results:**  24. I like the quotes being included within the text but it does make the whole paper very long. One strategy may be to place these in a table so that they don’t distract from the themes presented. Either that or if they could be made shorter that would also help. | 24. We focused once again on the quoted statements of the respondents. In those places where shortenings could have taken place without sacrificing text quality, we have eliminated some quotes. Thank you for this suggestion. Nevertheless, we could not include the quotes in a table because in our opinion this would make reading the manuscript much more difficult as it would mean the constant need to distract from the research results and refer to a table in which the reader would be forced to find the relevant fragment of the respondent's statement. |
| **Conclusions:** 25. These need to be included in the first part of the discussion.  26. I would remove the numbers from these paragraphs. They are not needed.  27. I would also attempt to rewrite these paragraphs in order to be more concise with your statements and to bring the word count of the paper down and so they provide a short summary prior to the more detailed discussion | 25. 26. 27. As suggested by Reviewer, 1 the form and content of the Conclusion part have been revised. Nonetheless, we would like to separate it from the Discussion and present a short summary of the research results based solely on the obtained data. We also would like to present this information in two separate perspectives: qualitative, concerning the experience of the body, and quantitative, concerning its image. This reflects the adopted research methodology. In the Discussion part, based on information from the literature on the topic, we integrate two types of data and try to interpret them. It is a deliberate procedure, also reflecting the interdisciplinary nature of the research and our cooperation. The surgeon, body sociologist and clinical psychologist worked on the project in concert but based on research tools specific to their field. The meeting, summary and reflection on the effects of the joint work are included in the Discussion. |
| **Discussion**  28. Page 29, paragraph 3: You mention that anxiety is a basic problem faced by donors. I would phrase this as it being a regular problem as basic implies that it is minor. I would also argue that you cannot conclude that it is primarily related to their body. There are a number of anxieties experienced by donors, primarily often related to their recipient.  29. You have also misquoted reference 62. This paper did not demonstrate that negative psychosocial difference disappeared over time. It showed that very little changed after donation when it came to a number of psychological factors. | 28. The remark about the anxiety experienced by the kidney donors seemed especially important to us. Indeed, narrowing the psychological problems faced by donors solely to anxiety is a significant simplification, even more so, ascribing fear only to matters of one's own physicality. Therefore, the content of the article was changed. In response to this remark from Reviewer 1, the source data was also supplemented with three new references.  29. Thank you for detecting inaccuracies in citing the publication sources. We have read the content of the source article once again. The effect of this is its removal from this part of the Discussion and the insertion of sources corresponding to the content. |

| **Response to comments from Reviewer No. 2** | |
| --- | --- |
| **Reviewer's comments** | **Authors’ replies** |
| 1. Regarding the title: I think the paper would benefit from slightly improving the title, as I feel that the second part of it “a report from interdisciplinary studies” does not reflect exactly the content of the paper. It gives to me at least the impression it is not an original study but a review; secondly the paper in my opinion describes complementary methods or mixed methods used to address the research question and appears as one study/project, so the plural „studies“ is confusing. | 1. Thank you for your suggestion to change the title. We trust that the current version more fully reflects its content. |
| 2. In the abstract, in results, the wording „reduced assessement“ feels unclear. | 2. Thank you for your comment. This information has been clarified. |
| 3. Line 76 „studies indicate no change in the overall quality of life“: this is an oversimplification and not a fair representation of the studies so far regarding QoL in LKD. | 3. We strongly agree with this remark. The indicated sentence has been changed into a more complete statement, supported by additional publications. |
| 4. Lines 90-92: the meaning of this phrase is not clear to me. | 4. Re-reading the part of the introduction that was criticized by the Reviewer indicated that: a. the sentence was in fact unclear; b. its removal does not change the main idea of this part of the work. |
| 5. The research questions is stated clearly at the end of the paper 832-834, but I think it would be good to mentioned so clearly also in the beginning where you state the aims of the study 146-151, as I think it will help the reader. | 5. The main research question was included in the Introduction section. Thank you for this comment. In fact, the formulation of the main research problem at this point allows us to follow our search for the theoretical foundations of the research (including the selection of publication sources), identify the specific objectives of the research, and also bring closer the decision regarding the selected research procedures. The question has been inserted before the detailed description of the objectives to make the text clearer. |
| 6. I am not sure I understand why the aim of the study is to search for effective and interdisciplinary research methods, (148-149) as you basically are already using them. | 6. Thank you for this extremely valuable comment. We have clarified the first research goal, which reflects our actual research intentions. |
| 7. Table 1. It would make sense to be more explicit regarding the donor-recipient relationship under related, unrelated (is it parent to child, spousesm friends? etc.). | 7. We explained what kind of relationship exists between the donor and the recipient in the study group. We supplemented this data with numbers. |
| 8. I understand it might be important to mention the types of surgery laparoscopic or open nephrectomy, but I find it exaggerated for this type of paper. It would make more sense to explain to the non-surgery affiliated reader the bodily implications or risks of these two types of nephrectomy in order to understand the impact of each, which I suppose is one of your aims. | 8. Thank you for this remark. We have resigned from the detailed description of the kidney donation procedure in favor of a simple and concise explanation of what this procedure is about, the differences between the two methods of kidney donation as well as the anatomical and physiological consequences of donation. |
| 9. The same goes for some other parts of the paper that I consider to be overly detailed, e.g. „the postoperative care of the donor“ 231-239 lines, 255-265. | 9. In the 2 fragments of the text indicated by the Reviewer, we have shortened the content so that it is not too detailed. |
| 10. Line 245: so who are these persons who carried out the study?? | 10. We supplemented the part describing the examination procedure with information about who conducted the research. |
| 11. The psychometric data were collected 6 months later. This is a problem and it needs tob e justified why and to state the limitations or strengths this approach and the time gap means. | 11. In response to the comment from Reviewer 2, we supplemented the content of the article in the part devoted to the examination procedure. We treat keeping the time gap between the sociological and psychometric parts of the research as a deliberate procedure, motivated by the need to maintain the separateness of two types of data: a. Qualitative data on experiencing one's own body; b. quantitative about his image. Conducting the research simultaneously could result in interaction of the conveyed content, e.g. through the occurrence of the priming effect. The interval between the quantitative and the qualitative part of the study was set at approximately 6 months. This time was assessed as optimal for the expiration of the memory trace of most of the interview content, and at the same time it did not significantly change the identification of one's body. |
| 12. This comment applies to the whole paper but reading lines 286-290 made me ask myself even more about it, why is this study or at least the qualitative part a sociological one? The dimensions you include in the interview outline lines 286-290 seem more psychological to me . The same applies later on for the results, I have difficulties identifying the sociological character of it when speaking about coping, adaptation, perception, terms that in my experience at least belong more to the field of psychology. I understand sometimes boarders between disciplines are not clear, and I could place the study in the field of social sciences in general and not sociology necessarily. Connected to this, I understand the study more as a mixed methods approach study and less interdisciplinary in this way, but you can argue about that. | 12. In response to comment 12 from Reviewer 2, allow me to enter into a discussion. The presented research, in its sociological part, is sociological research, as evidenced by:   1. The research methodology - the researcher consistently uses the methodology of the grounded theory in its sociological version (Kathy Charmaz's social constructivism). 2. The theoretical framework of the conducted research, which is based on such sociological theories as symbolic interactionism (Anselm L. Strauss) and social phenomenology (Maurice Merleau-Ponty, Alfred Schütz). 3. The interpretation of the research results using sociological categories and concepts specific to the language of the discipline.   For the above-mentioned reasons, the presented sociological research cannot be called psychological, nor can it be generally presented as research in the field of social sciences.  The dimensions of sociological research on the experience of the body by kidney donors (1. somatic; 2. adaptive; 3. socio-interactive; 4. identity) presented in the text are obviously not those issues that can only be investigated using sociological methods. I agree that they can also be successfully researched by a psychologist. Nevertheless, a sociologist will examine and describe them differently than a psychologist. They will reach for different research methods, but they will also use different frames of reference, which are the resources of theoretical knowledge specific to these disciplines. The sociologist carrying out this part of the research, being a qualitative sociologist, assumed the role of a meaning seeker and focused on understanding the meaning of nephrectomy in the context of experiencing one’s own body by the donors and the impact of this interference with the body on the self-conceptions and personal identity of the donor kidney. In exploring the identity consequences of donation, the researcher, as a sociologist, focused on the socio-interactive embedding of an individual's identity.  Our research, in its sociological part, is not pioneering research on the body in social aspects. There is a whole separate field of sociology called sociology of the body that arose in the mid-1980s. The sociology of the body is a sociological subdiscipline with a well-established cognitive and scientific identity, as evidenced by the multitude of scientific works published in this field. As an example, I will give the textbook *The Body and Society. Exploration in Social Theory* (1984) by Bryan S. Turner, who is considered to be the founder of this field of sociology, and the peer-reviewed journal *Body & Society*, published since 1995 (editor Mike Featherstone), which publishes texts on key topics in the field of body research. Common to theoreticians and researchers in the field of the sociology of the body is the thesis that human identity is rooted in the physical sphere. Therefore, all the above-mentioned dimensions of researching the experience of one's own corporeality have been and are studied by body sociologists and medical sociologists (especially in the interpretative trend), as exemplified by numerous works devoted to the relationship of the body and identity in the context of health, disease or the development of medical technologies interfering with the body. Finally, I will recall examples of such works:  Charmaz K. Loss of Self: A Fundamental Form of Suffering in the Chronically ill. Sociology of Health and Illness. 1983; 5: 168-195.  Charmaz K. The Body, Identity, and Self: Adapting to Impairment. Sociological Quarterly. 1995; 36: 657-680.  Charmaz K. Good Days, Bad Days. The Self in Chronic Illness and Time. New Jersey: Rutgers University Press; 1997.  Nettleton S. The Sociology Health and Illness. Cambridge: Polity Press; 2006.  Nettleton S. „I Just Want Permission To Be Ill”: Towards a Sociology of Medically Unexplained Symptoms. Social Science and Medicine. 2006; 62: 1167-1178.  Yoshida K. Reshaping of Self: A Pendular Reconstruction of Self and Identity Among Adults with Traumatic Spinal Cord Injury. Sociology of Health and Illness. 1993; 15: 217-245. |
| 13. The methods are described in a very detailed manner. This increases understanding and transparency but it feel sometimes a more condesned presentation would leave more space for more discussion. | 13. The description of the qualitative methods has been shortened so that the contents remain in line with the criteria for reporting qualitative research on the COREQ list recommended by PLOS ONE:  <https://journals.plos.org/plosone/s/submission-guidelines#loc-qualitative-research>  <https://academic.oup.com/intqhc/article/19/6/349/1791966> |
| 14. I do not understand line 339. | 14. We explained that in writing "naturalized transcription" we mean a copy of spoken discourse. |
| 15. The interview outline is missing and I think it should be added in a table or annex. | 15. As the mere outline of the interview might not be sufficient, in Appendix 1 we have presented a complete list of the researcher's information needs, which includes both general and open-ended questions as well as specific and structured questions the respondents were asked in order to explore particular threads of the conversation. We decided that such a formula would allow the Reviewers and Readers to get to know the interview scenario better. |
| 16. Lines 420-422. Who collected the psychometric data? | 16. Information about the person carrying out the psychometric tests has been supplemented in the text. Thank you for noticing this important deficit. |
| 17. My major comment regarding this paper has to do with the presentation of the results in connection with the theoretical sampling you mentioned you applied. You say you applied theoretical sampling and included in your sample non related donors and donor from paired exchange donation. Yet the results do not mention anything related to this aspect, whether it makes a difference or not. I think this should be addressed. Does it not make any difference and if not why? in the discussion Furthermore, it is a pity you could not include during your theoretical sampling more different or extreme cases of donors who experienced complications, or their kidney was rejected or the recipient died or had complications. This makes the results appear a bit flat and less complex as they might be, and they „beautify“ or simplify the experience, as the way the donors experience their body and body image is embedded and it depends possibly on the different relationships and contexts and postoperative courses, complications etc. There are reports of donors experiencing fatigue after donation which is the opposite of the highly functioning body you mention. It would have been interesting to include such donors in your sample to broaden the „grounded theory“ derived from the data. I understand this is not possible now and maybe even not the point. Yet, in this case I think you should mention all these aspects in the discussion and in a part called limitations of the study. Otherwise there is the danger that your results are adopted uncritically by clinicians or others to promote specific interests, as your results reflect only a part of the phenomenon living kidney donation and this should be made clear that this is a sample of successful donations. | 17.  A. Thank you for your remark on the importance of kinship in the way donors experience their own body. In the presentation of the results and in the conclusions from the research, we wrote that the relationship of kinship was irrelevant to individual aspects of the donors' experience of their own corporeality. The analysis of each of the research threads confirmed the lack of importance of the kinship between the donor and recipient for the way the subjects experience their own body. Furthermore, the discussion was expanded to explain why kinship is irrelevant to the way one's body experiences after donation.  B. We consider this remark extremely valuable in the context of further sociological research on the experience of their own body by kidney donors. Notwithstanding, this will require a reformulation of the research project and obtaining a separate consent from the bioethics committee for the inclusion of donors with complications or those whose kidneys were rejected by the recipient's organism or the recipient died. Such an extension of the study group will certainly enrich the theory generated from the research conducted so far. Thank you very much for this remark, which has inspired us to carry out further research projects on the phenomenon of living donation.  C. We consider the comment to be one of the most important and key changes in the text. In fact, the tone at the end of our article would only indicate positive donor experiences. We agree (based on the extensive literature on the subject and our other research) that this is not true. We strongly agree that we could obtain completely different results in groups experiencing significant post-donation difficulties (both their own and the recipients). Therefore, in the part of the article devoted to research limitations, we made a change. Hopefully, this will be a clear indication that no hasty conclusions can be drawn from the obtained results. The article is absolutely no voice in the "for versus against living donation" discussion. It presents facts, and the observance of scientific rigors and the transparency of the results allows it to be treated as an important voice in the scientific discussion.  We also wanted to explain the effect of the lack of people who experience serious consequences of donation (their own or indirect recipients) in the group of respondents.  The subjects were recruited to the project in accordance with the rigors and principles of the grounded theory method. Until the theoretical sampling method was applied, as a result of which donors unrelated to the recipient were included in the study, the properties of the central category of the study (experiencing one's own corporeality) were uniform. This prompted the researcher to ask whether this category could be differentiated by the lack of kinship between the donor and recipient. Following the logic of theoretical sampling, the researcher sought here to discover differences within the analyzed analytical category, i.e. within the process of experiencing their own body by donors. Although this direction of analyses did not confirm the researcher's assumptions, it significantly expanded the analysis, in particular, by including unrelated donors in the research, another important analytical category was developed that had already emerged from the theory, which was "extended bodily identity". It turned out that donors experience a broadened body identity also when the recipient is a stranger to them. Thus, it is not the kinship that causes this state, but the effective control of the body, the meaning of which is to maintain the unity of the bodily identity. To sum up, the failure to include donors experiencing their own complications or the recipient's complications in the study group was a consequence of the direction of analytical work chosen by the researcher.  We are aware that such a selection of the studied group limits the possibility of a full answer to the research question, narrowing down the picture to well-functioning donors. The results of our research to date allow us to extend the research to include groups of donors indicated by the Reviewer. This is our intention in the near future. This project, as already mentioned, will, however, require significant changes. Firstly, it should take into account an additional variable, which will be medical complications or psychological trauma. Secondly, with such a sample selection, we face logistical challenges, including obtaining the necessary consent of the respondents and not interfering with the procedures of the study in the process of treating patients. Third, we need to obtain the Ethics Committee's approval again to research a group of donors experiencing complications directly or indirectly. Fourthly, the technique of conducting in-depth interviews must be changed because the interview with a traumatized person should be additionally secured with psychological support. |
| 18. It would help to add in annex the questionnaire you applied, to help the reader understand the psychometric part of the study and the result better. | 18. The Body Esteem Scale, adapted by Małgorzata Lipowska and Mariusz Lipowski, is a tool open to the public in Poland. Nevertheless, we do not have permission to make its original version available in scientific publications. In the text of the article, though, we provide the publication source regarding the questionnaire. It describes not only all the questions of the questionnaire, but also the entire procedure of adapting this tool. In accordance with the principle of transparency, I am sending the database on which the statistical analyses were performed. The database contains a record of all the questions in the questionnaire. Responding to the reviewers' comments, we added sample questions to the description of the tool for each subscale, as well as for both sexes. |
| 19. Unfortunately the study is not prospective so the statements of the donors about their attitude towards their body before donation is difficult to accept. | 19. Thank you for this comment. We agree that our research is not prospective. Therefore, in response to this remark, we have removed from the research results, conclusions and abstracts all those passages that pertain to how donors experienced their own body before donation. |
| 20. The hypothesis you developed based on the qualitative part and tried to test through the quantitative are not very clear. It think the part lines 767-824 needs to be presented more clearly. | 20. Thank you for pointing out the lack of clarity of the information provided. The descriptions of the hypotheses and analyses have been corrected. |
| 21. Also when you say that some body parts are rated lower it is not clear to me exactly what lower means. e.g. line 857. | 21. Thank you for this remark. In fact, merely identifying a “worse” assessment of the body parts directly related to the procedure may not be sufficient. Therefore, both in the abstract and in the section devoted to the conclusions, this information has been clarified. A poorer rating means receiving results that indicate a negative emotional evaluation of the body part in question. |

*We hope that the above answers correspond to all the comments submitted by the reviewers.*

With best regards,

Corresponding author:

Katarzyna Kowal, PhD

Jan Dlugosz University in Czestochowa

ul. Armii Krajowej 13/15

42-218 Czestochowa, Poland

k.kowal@ujd.edu.pl
