# Supplementary material for: Experiencing one’s own body and body image in living kidney donors–A sociological and psychological study
Source: PLoS One. 2021 Apr 15;16(4):e0249397. doi: 10.1371/journal.pone.0249397 (PMC8049271; doi:10.1371/journal.pone.0249397)
Supplement: Attachment — Submitted filename: Response to Reviewers.docx [file pone.0249397.s004.docx]

22 January 2021

**Rebuttal letter**

Manuscript Number: PONE-D-20-15970

Full title of submitted article: Experiencing one's own body and body image in living kidney donors – a sociological and psychological study

Order of Authors: Katarzyna Kowal (corresponding author), Mateusz Zatorski, Artur Kwiatkowski

*Thank you for the subsequent thorough and substantive evaluation of our article. We would also like to thank you for the words of praise expressed towards our work at the previous stage of the review procedure. This time, we have made every effort to respond to the submitted comments as honestly as possible.*

*We present below the authors' responses to the comments of both Reviewers. They concern both linguistic errors and inaccuracies as well as those in the substantive layer of the text. In order to present them in the most orderly and legible way, we have included them in the table below.*

| **Response to comments from Reviewer No. 1** | |
| --- | --- |
| **Reviewer's comments** | **Authors' replies** |
| **Abstract**  1. Results: “lack of kidney in the body” would read better as “absence of a kidney in the body”  2. Conclusions: final sentence is very wordy. May read better as: The proposed approach utilising mixed methodology within the fields of sociology and psychology for researching the phenomenon of living kidney donation is definitely innovative. | *1. Thank you for this linguistic comment. We agree with this suggestion. The change has been applied.*  *2. Thank you for proposing a better wording of* *this sentence. The change was introduced and combined with Reviewer No. 2's comment.* |
| **Main Text**  3. Line 74 – “… negligible risk of physical and mental health disorders for living kidney donors”  4. Line 170 – The first concern that needs to be taken into account is the highly subjective… | *3. and 4. Thank you for pointing out the lack of precision in the terminology used in lines 74 and 170. We have corrected the indicated phrases after re-examining the cited literature.* |
| **Table 1**  5. Please clarify what is meant by “permanent / no permanent relationship” | *5. Thank you for drawing attention to this vague sociodemographic category. It has been renamed and assigned clearer subcategories as well.* |
| 6. Line 213: should read “symphysis pubis”  7. Line 214: shorter pain is incorrect. Should read ‘pain is less’ or ‘pain is reduced’  8. Line 237: consent being obtained ‘verbally’ is better English  9. Line 320: should read “were more likely” (past tense) | *6., 7., 8. and 9. We agree that the suggested language phrases and forms are more correct in the English language. All the corrections have been made. Thank you.* |
| 10. Line 333: Would read better as ‘None of the invited participants declined to participate in the interview, nor ended it prematurely’ | *10. Thank you for the better wording of this sentence. It has been applied.* |
| **Conclusions**  11. Rather than use the word ‘goals’ I would refer back to the aims for the study – ‘Both of the aims of the study have been/were achieved’  12. I’m unsure what ‘project realization’ is | *11. We have made the suggested changes to this sentence, which makes it more precise. Thank you.*  *12. We used the phrase 'carrying out the study'. We trust that in this way the meaning of this sentence is clearer. Thank you for making this comment.* |

| **Response to comments from Reviewer No. 2** | |
| --- | --- |
| **Reviewer's comments** | **Authors’ replies** |
| 1. The paper still feels too long and there is a lot of repetition mostly when it comes to the description of the method (especially grounded theory and the sociological background of the author). As much as I admire the passion of the first author regarding the methods, they way it is written makes it at least for me difficult to follow sometimes due to repetition, on expense of the results. Thus, I would recommend the authors identify repetitions and re-write in a more condensed manner. | *1.* *The article was shortened in the 'Introduction' and 'Qualitative methods' parts. First of all, we shortened the threads concerning the description of the grounded theory methodology as well as the motives for its application in the study. We removed content redundancy. Thank you for this comment. We hope that the reading of the text will now be easier.* |
| 2. Even though I am not a native english speaker, I still think there are some formulations that do not feel right. I am unable to highlight those throughout the text, as I am not an expert on this, but just a small example in Table 1 "permanent and not permanent donor-recipient relationship" . I do not understand this term and I cannot imagine what it means. Or Line 336 "general interview scenario". I think you mean interview outline? | *2. Thank you for drawing attention to this vague sociodemographic category. It has been renamed and assigned clearer subcategories as well.*  *As for line 336, in fact, we meant interview outline. Thank you for pointing out this linguistic inaccuracy.* |
| 3. In the abstract the last few lines "The proposed research approach consisting in the use of mixed methodology in the fields of sociology and psychology in research on the phenomenon of living kidney donation is definitely innovative" need to be changed. Even though the use of those methods are not very common in living organ donation, they cannot be presented anymore as "definitely innovative". I consider this an exaggeration (and I am doing such studies myself) and suggest to replace it with another adjective. | *3. We agree that the use of a mixed methodology in sociology and psychology in studying the phenomenon of living kidney donation is not innovative, it is simply not very common. Thank you for this remark.* |

With best regards

Corresponding author:

Katarzyna Kowal, PhD

Jan Dlugosz University in Czestochowa

13/15 Armii Krajowej St.

42-218 Czestochowa, Poland

k.kowal@ujd.edu.pl
