# Supplementary material for: Experiencing one’s own body and body image in living kidney donors–A sociological and psychological study
Source: PLoS One. 2021 Apr 15;16(4):e0249397. doi: 10.1371/journal.pone.0249397 (PMC8049271; doi:10.1371/journal.pone.0249397)
Supplement: Attachment — Submitted filename: Response to Reviewers.docx [file pone.0249397.s005.docx]

15 March 2021

**Rebuttal letter**

Manuscript Number: PONE-D-20-15970

Full title of submitted article: Experiencing one's own body and body image in living kidney donors – a sociological and psychological study

Order of Authors: Katarzyna Kowal (corresponding author), Mateusz Zatorski, Artur Kwiatkowski

*Thank you for the subsequent thorough and substantive evaluation of our article. This time, we have made every effort to respond to the submitted comments as honestly as possible.*

*We present below the authors' responses to the comments* *from Reviewer No. 1.*

| **Response to comments from Reviewer No. 1** | |
| --- | --- |
| **Reviewer's comments** | **Authors' replies** |
| Line 74: The data from studies carried out by various transplant centers on a large group of donors indicate a low risk of complications during and after the donation procedure [1].  This should read: The data from studies carried out by various transplant centers on large groups of donors indicate a low risk of complications during and after the donation procedure [1]. | *Thank you for the better wording of this sentence. It has been applied.* |
| Line 174: The first one concerns emphasizing the subjective nature of experiencing one's own body and the changes taking place in it [37].  This should read: The first one concerns emphasizing the subjective nature of experiencing one's own body and the changes taking place within it [37]. | *Thank you for this linguistic comment. We agree with this suggestion. The change has been applied.* |
| Table 1: The marital status still does not make sense and requires further clarification. You have categorised people as either married or single / and those not in permanent partnership relationships. I would either categorise these as 'married' and 'single' or you need to say 'married / in a long-term relationship' and 'single / not in a long-term relationship'. This will the provide a clear distinction between those two groups. | *Thank you for drawing attention to these vague sociodemographic categories. The changes have been applied.* |

With best regards

Corresponding author:

Katarzyna Kowal, PhD

Jan Dlugosz University in Czestochowa

13/15 Armii Krajowej St.

42-218 Czestochowa, Poland

k.kowal@ujd.edu.pl
